# Kidney Sellers From a Village in Nepal: Protocol for an Ethnographic Study

**DOI:** 10.2196/29364

**Published:** 2022-02-24

**Authors:** Bijaya Shrestha, Bipin Adhikari, Manash Shrestha, Luechai Sringernyuang

**Affiliations:** 1 Department of Society and Health Faculty of Social Sciences and Humanities Mahidol University Nakhon Pathom Thailand; 2 Mahidol-Oxford Tropical Medicine Research Unit Faculty of Tropical Medicine Mahidol University Bangkok Thailand; 3 Contemplative Education Center Mahidol University Nakhon Pathom Thailand

**Keywords:** ethnography, kidney selling, Nepal, qualitative methods, study protocol, bioethics, medical ethics, kidney transplantation, living donors, tissue donors, tissue and organ procurement, transplantation, organ transplant

## Abstract

**Background:**

Kidney selling is a global phenomenon, with higher-income countries functioning as recipients and lower-income countries as donors, reflecting the gaps due to poverty and vulnerability. In recent years, an increasing number of residents in a village near the capital city of Nepal have been selling their kidneys; however, the factors embedded in the local social, cultural, political, and individual context driving kidney selling are poorly understood.

**Objective:**

The aim of this study is to explore the drivers of kidney selling and its consequences in Hokse village in central Nepal, using ethnographic methods and multistakeholder consultations.

**Methods:**

An ethnographic approach will be adopted along with in-depth interviews and key informant interviews among the residents and kidney sellers in the village. Relevant participants in the village will be selected purposively using a snowball approach. The number of participants will be predicated on the principles of data saturation. In addition, consultations with relevant stakeholders will be conducted at various levels, which will include authorities within and outside the village, and policymakers. All interviews will be conducted face to face, audio-recorded for transcription, and subjected to a thematic analysis.

**Results:**

This study was approved by Mahidol University Central Institutional Review Board (MU-CIRB 2020/217.1808) in September 2020 and by Nepal Health Research Council (NHRC 716/2020 PhD) in January 2021. The fieldwork started in February 2021 and the data analysis was completed in September 2021.

**Conclusions:**

This study is expected to provide insight into the reasons underlying the practice of kidney selling based on the example of Hokse village, along with the perspectives of multiple stakeholders.

**International Registered Report Identifier (IRRID):**

DERR1-10.2196/29364

## Introduction

The increasing global demand for kidney transplantation has fueled an illegal kidney trade. In 2011, 10% of all organs for transplant surgeries in the world were trafficked from a total of 100 countries [1,2]. The global phenomenon of the kidney trade finds major buyers in high-income countries and sellers in low- and middle-income countries (LMICs) [3,4]. Kidney selling has been identified in places such as the “shantytowns” of Brazil, which are infamous for “body snatching” [5], and various other regions such as in South Africa, the Philippines, Israel, Turkey, Moldova, Argentina, Mozambique, Eastern Europe, Egypt, and India. Often, the poor and vulnerable communities in these LMICs are the hotspots for the kidney trade. In Asia, the kidney trade is prominent in China and the Indian subcontinent. Although economic prosperity is rising in these countries, high inequality between the rich and poor is pressing hard on the population. Thus, the poorer segment of the population continues to fall vulnerable to the easy money that can be obtained by selling their kidneys, while being unaware of its adverse consequences [6]. However, little systematic literature exists in exploring the nature of the organ trade, its drivers, and the adversities.

Nepal, a small country sandwiched between China and India, has become notorious for an increasing kidney trade in recent years. The village of Hokse, which lies approximately 50 kilometers east of the capital city, Kathmandu, has a high proportion of villagers who have sold their kidneys. In 2015, a study reported that more than 300 villagers from Hokse had already sold their kidneys for as low as US $200 [2], most of whom had been deceived by brokers with promises of a better future and substantial financial rewards [7]. Consequently, the village has been labeled as the “kidney village,” which serves the cheapest kidneys in the world [3]. In addition, two massive earthquakes in 2015 further pushed the villagers of Hokse to extreme poverty and compounded their existing vulnerabilities for kidney selling. An immediate impact of the earthquakes was that the demolished houses needed urgent repair or rebuilding for which some villagers resorted to selling their kidneys [8]. Given the proximity to the capital city and the good road connection, villagers could seek jobs in the city for their livelihood. These social and cultural circumstances further add to the urgency and interest in focusing on Hokse village as a case study for the kidney trade.

Although poverty and economic conditions may seem to be ostensibly prominent reasons for kidney selling, they are invariably entrenched in the local social and cultural context such as money for food, rituals, and dowry [6,7,9-17]. Kidney sellers are often left with an even worse financial situation and physical health after kidney removal [4,6,9-11]. Thus, a deeper analysis of the phenomenon and its drivers, along with the underlying links to the wider structural, social, and cultural factors in the village, is essential. This study was conceived to fully explore the phenomenology in Hokse village, in addition to answering how it differs from other villages that share similar social, economic, and cultural contexts.

The main objective of this study is to explore the drivers of kidney selling and its consequences in Hokse village of the Paanchkhal municipality in central Nepal, using ethnographic methods and multistakeholder consultations. The specific objectives of this study are to: (1) understand the nature and pattern of the kidney trade in the community; (2) explore Hokse residents’ perception, concept, and meaning of the kidney and the kidney selling phenomenon; (3) explore various factors driving the kidney trade from multiple stakeholders and actors such as village head leaders, neighbors, and family members; and (4) explore the enabling and impeding factors related to the kidney trade in and beyond Hokse village.

## Methods

### Study Design and Setting

As a qualitative research design is deemed to be the most appropriate for exploring and understanding the concept of kidney selling, the meaning of the kidney, and the interplay of myriad factors affecting the kidney trade [18], we will employ different qualitative techniques and utilize Hokse village as a case study. Multiple methods of qualitative data collection will be used, including an ethnographic approach in Hokse village and in-depth interviews with kidney sellers and other relevant participants.

The study setting, Hokse village, is located at ward numbers 7 and 8 of Paanchkhal municipality, Kavrepalanchwok district, which is about a 2-hour drive from Kathmandu. There are a total of 1000 households in these wards [19]. The members of Hokse village include different ethnicities such as Bahun, Chettri, Tamang, Sarki, and Danwar, and their livelihoods depend on subsistence farming and daily wage-earning [19]. Given the proximity to the capital city and a good road connection, a natural question arises as to why the villagers choose to sell their kidneys rather than seeking economic opportunities in the capital city.

### Study Population and Sampling

Data will be collected from a diverse group of participants. The primary participants for the research will be kidney sellers aged above 18 years (Table 1). In addition, family members, neighbors, relatives, village heads, and permanent residents of the village who are willing to discuss the issue will be included as key informants of the study. Health care providers and local officers of different developmental organizations will also be recruited as our key informants. Other stakeholders such as policymakers at the Ministry of Health, legal workers, and civil society organization staff working in the field of organ and human trafficking for at least 1 year will be included in the study. Similarly, medical personnel involved in transplant medicine in Nepal will be interviewed. We will also interview some border patrol officers posted at an important border checkpoint for at least the previous 6 months. Data from these different types of participants will allow us to gain a holistic view of the phenomenon. However, we will not include altruistic kidney donors or those who undergo the surgery to donate to their family members.

Informed consent will be obtained orally and the prospective participants will be requested to sign the consent form after receiving information about the study. Informed consent in this study will be undertaken thoroughly. For example, informed consent among kidney sellers would be a sensitive issue, and thus all measures for complete and confidential informed consent will be taken into consideration.

A notable aspect of this study is the ethnographic fieldwork in which a researcher will familiarize with the villagers by living in the villages, and will observe and note down the life, culture, and traditions in the village. Such a method does not enable obtaining consent from potential participants as would be done for a survey or qualitative interview. Nonetheless, at minimum, the researcher can obtain informed consent for all types of interviews. In case of virtual interviews, the researcher will obtain oral consent along with a digital signature of informed consent. However, some of the conversations that occur in a causal fashion as part of everyday life would be considered informal interviews and may not require informed consent.

The primary participants will be given full authority to choose the interview site and decision to be interviewed. An interviewee will be clearly instructed that they can discontinue the interview at any point without providing any justifications. All participants will be anonymized in the collected data and all personal identifiers will be removed from the transcripts.

During the study, a series of purposive sampling efforts will be made to identify the various participants. The purposive sampling technique will allow us to identify and collect information-rich cases for an in-depth study [20]. As the issues around the kidney trade can be culturally sensitive, a snowball approach may be used to identify additional participants until data saturation is reached. However, the sampling design may evolve depending on the circumstances of the data collection period, the opportunities to enroll the relevant participants, and the ability to capture the issues that emerged during the stay in the village. Specifically, we will interview approximately 12 to 20 kidney sellers, ensuring an adequate sample to obtain varied stories of kidney selling [20-23], along with 18-28 key informants and stakeholders (Table 1).

**Table 1:** Details of the study population, participants, and sample size.

Type of participant		Sample size, n
Primary participants: Kidney sellers of Hokse village		12-20
**Key informants**		
	Family members, neighbors, relatives, village head	10-15
	Health care providers and local officers	2-3
**Stakeholders**		
	Transplant unit medical personnel	2-3
	Policymakers, legal workers, NGO^a^/INGO^b^ workers	3-5
	Border checkpoint officers	1-2

^a^NGO: nongovernmental organization.

^b^INGO: international nongovernmental organization.

### Research Tools

We will use interview guides for the in-depth interviews and field diaries for the ethnographic study (see Multimedia Appendix 1 for the interview guide). The field diary will be used to record important details of the ethnographic observations such as nonverbal communication, including the personal presentation of the participant, body expressions, gestures, facial expressions, style, and alterations in speech (eg, silences, choking speech, blatant speech, fading speech, cringing and tremors in the speech), laughter, and other manifestations. The principal investigator (BS) will be an integral component of the research who will investigate how local people think, perceive, and justify the kidney selling phenomenon.

Furthermore, every attempt will be made by the researchers to understand the participants and remain at the same level during constructing the meaning and experiences of kidney sellers [24]. This will help to interpret the discussion as shared by the participants. The preliminary conception that kidney selling was highly prevalent in the village to fulfill the basic needs of the poor villagers is incomplete, particularly as there are poorer villages without a kidney trade in Nepal. Understanding the experiences of kidney sellers will be critical in building an emic meaning and concept of the kidney and its trade.

### Researchers’ Background

The characteristic of the researchers is an important aspect to consider for the research design, data collection, and analysis, as it may have an impact on the participants. The principal investigator (BS) has an educational background in health and social sciences and belongs to a Newar ethnic community in western Nepal, where he has lived near different ethnic groups such as Bahun, Chettri, Magar, Gurung, Darai, and Bote, which is similar to the cultural context of Hokse village [25]. Although all team members of this study are men, this team is well trained to tackle the sensitivity of the research. The team members include an expert medical anthropologist (LS) and a public health specialist (BA) who have expertise and experience around community engagement, along with a researcher (MS) who has experience in gender-related issues. We believe that this research team has the capacity to think critically and add multidisciplinary perspectives to explain the unanticipated circumstances to enrich the data interpretation and its implications. Based on the ongoing data collection and reflection, we will train and incorporate a woman to collect data as to better reflect gender-related issues and when approaching female participants.

### Research Hypothesis

The organ trade in Hokse village is a multifaceted problem that is predicated on individual microlevel to macrolevel factors such as policy and geographical context. This study will attempt to explore this hypothesis using a combination of methods, primarily through ethnography.

**Figure 1:**
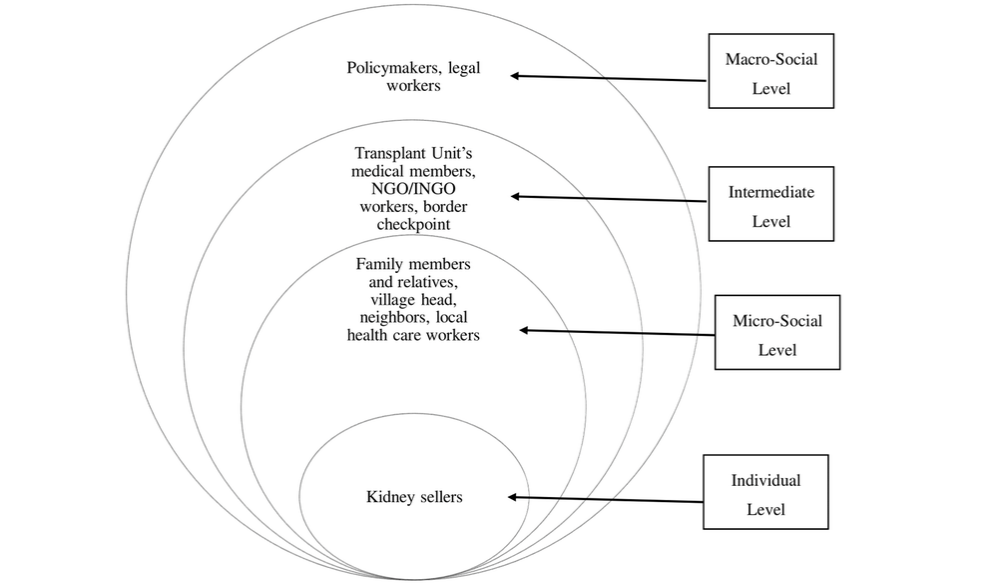
Participants, key informants, and stakeholders at different levels. NGO: nongovernmental organization; INGO: international nongovernmental organization.

The organ trade is not a trade in the conventional sense; it is the selling of human flesh. Thus, its presence in any society is not a simple phenomenon. As kidney selling is a complex phenomenon, its research warrants a flexible approach. We will use a constructivist perspective, which is one such approach in which human beings are assumed of being able to construct their understanding of the subject and situation. We will also analyze the embedded class and power relations, social inequalities, and social interactions that shape individual decision-making. Using the theoretical lens of critical medical anthropology (CMA), we will delve deeper into the experiences of the kidney sellers to understand and situate their decisions with the power differences and influence of dominant sociocultural and economic forces. Multiple stakeholders and key informants will allow us to understand the web of causation of kidney selling at different levels of analyses as used in CMA, from the individual microlevel to the macro structural level (Figure 1) [26]. CMA highlights the importance of political and economic forces, including the exercise of power in shaping health, disease, illness experience, and health care, which supplements the culturally sensitive analysis of human behavior grounded in anthropological methods.

### Data Collection Procedures

#### Entry into the Field

Before drawing up this research protocol, the principal investigator (BS) visited the village twice to explore the feasibility of the study. In the first visit, a few kidney sellers were located with the help of some personal contacts. In the second visit, some key informants were identified and a further network was established. The experiences and connections made in these visits will facilitate a smooth integration into the village for the principal investigator, who will reside there during this research for ethnography. This approach will set a good rapport with the villagers and informal conversations will be conducted with the villagers before diving more deeply into the sensitive topic of kidney selling.

Although we intend to mitigate our preexisting biases before entering the field, our backgrounds and experiences, which are different from those of the villagers, may lead to some inevitable biases. It will be difficult to enter the field with an empty mind as the theoretical knowledge and literature will prompt the researchers to have a preconception [24]. Nevertheless, we will utilize an inductive approach, which is likely to uncover new ideas and observations, giving rise to new knowledge; as a result, new theoretical positions may be derived from our empirical data and observations [27].

#### Ethnographic Approach

##### Fieldwork

We will conduct at least 3 months of ethnographic fieldwork in the village. Even though the principal investigator (BS) is from Nepal, spending substantial time in the village is crucial to embed in the community. The fieldwork will be conducted based on the theoretical framework to address the objectives of the research. During fieldwork, observations will be the key tool to understand the different activities of the villagers. New questions and issues are bound to arise during the ethnographic research. The research methods will be adapted to ensure that there is sufficient flexibility to gather crucial details during the ethnography. In addition, we will attempt to understand the power differences and interconnectedness of indigenous health and western medicine during our data collection, which can help us to explain and interpret the data [28]. Because of the current COVID-19 pandemic, we will adopt all national and local regulations, in addition to undertaking the safety and hygiene measures such as regularly washing hands, using alcohol gel, wearing masks, and social distancing. We will also share our knowledge (related to precautions and preventive measures for COVID-19) to our participants and will not remain in direct physical contact with any participants.

Concerning the risk management related to potential for stigmatization, since a researcher will be based in the village, we will utilize complete confidentiality when setting up interviews and discussion on the topic. No interviews will be openly conducted. An interview will be conducted in a quiet, safe, and enclosed place such as household rooms where others will not be allowed to participate. We will adopt in-depth interview techniques to ensure we have one-on-one interviews.

Considering the current COVID-19 pandemic, we will adjust our plans for data collection as feasible. For instance, we will hire a local Nepali as a research assistant to conduct the ethnographic study. Outside the village, interviews with stakeholders will be conducted remotely through, for example, Skype, Microsoft Teams, and Zoom. All coordination and management of data collection will be remotely managed by the principal investigator (BS) because of current travel restrictions due to the COVID-19 pandemic.

##### Participant and Key Informant Interviews

Iterative interviews will be conducted with the kidney sellers and their relatives, key informants, local leaders, and concerned people. The interview guide will be used flexibly to adapt according to the contextual situation of each unique participant. The saturation of data will be ascertained after timely discussions with the coinvestigators. As kidney sellers may have experienced harsh social stigma, they may not open up easily when interviewed. The principal investigator will be cognizant of the sensitivity and approach the challenge with empathy and patience. Only after an adequate relationship has been built with the kidney sellers, the principal investigator will attempt to explore the sensitive topics. The use of a snowball approach may be needed to locate further relevant participants based on the understanding that a particular participant might be key to the research topic [29].

#### Stakeholder Consultations

Different stakeholders will be consulted via semistructured interviews to understand the other actors of the research, as identified in Table 1. The main purpose of involving these stakeholders is to place the experiences of the kidney sellers in the social, cultural, political, and economic context of Nepal. For example, most kidney sellers travel to India through many of the checkpoints on the 1600-kilometer-long porous border that Nepal shares with India in the east, south, and west, where no legal documents are required to cross to the other side. To gain a better sense of how kidney sellers cross the border, the research team will travel to the Nepal-India border and interview the responsible personnel working at the checkpoint. Additionally, considering the COVID-19 pandemic, if stakeholder interviews are not possible as planned, we will request virtual interviews according to the preference of the interviewee.

#### Researchers’ Reflection and Discussion

After the interviews are conducted, the principal investigator will share the preliminary findings with the research team for reflection and further discussion. Based on the team’s discussion, the principal investigator may have to further explore particular themes. There may be multiple rounds of discussion and reflections among the investigators. As this research is a form of interpretive ethnography, the chances of revisiting the participants will remain open during the data collection period.

#### Rigor in Qualitative Data Collection

To ensure the information in qualitative research is reliable and valid, social interaction and trust between the researcher and participants are essential. We will attempt to avoid any bias by being reflexive, separating personal interpretations from the tasks during data collection and interactions with participants, and focusing more on developing an emic perspective [30]. To ensure that the study findings are shaped by the participants and not by researcher biases and interests, a trail of the research steps taken will be documented in the field diary. There will also be constant validity checks by checking for consistencies and inconsistencies in the data reported by participants. Alternative descriptions and explanations will be sought and recorded in the field diaries. Persistent observations during multiple sessions of interviews with different participants will help to triangulate the data and boost the credibility. Furthermore, a coinvestigator (BA) is an expert in the field of kidney selling in Nepal and will help to assess the credibility of the data extracted from the participants. Similarly, a particular focus will be maintained to adhere to the research guidelines and objectives of the research. The field diaries, transcribed data, and themes developed will be reviewed by two qualitative methods experts (LS and BA) to ensure that the interpretation and conclusions drawn are supported by the data.

### Data Analysis

The analysis of the data will start from the early phase of the research. The research methods used in this study will require different analyses. For instance, different facets of human experience, beliefs, kinship patterns, and ways of settlement and living, along with social, economic, and cultural factors of the village will be analyzed from the ethnographic findings. The audio-recorded interviews and the field notes will be transcribed first in the Nepali language and then translated to English. The translated English data will be back-translated into the Nepali language to ensure the validity of the translation, which will also help us to retain the nuanced meanings and interpretations. The interview data will be sorted and labeled. The data will be read several times to get a gist of the interviews, and then will be subjected to initial coding, followed by theme-building. The data will be analyzed and interpreted using the lens of CMA.

Content analysis will be conducted independently by two investigators (BS and BA). The initial analysis will be discussed with the participants to ensure that their emic interpretation is retained. NVivo version 10 (QSR International) will be used to manage and analyze the data. The analysis process and findings will be shared with the research team.

### Ethics and Dissemination

All participants will be provided with a complete and clear explanation about the research in the local language through an informed consent procedure. The participants will be made aware that their names will not be written anywhere in the document. Their participation will be completely voluntary and they will be clarified about their freedom to opt out of the study at any stage of the research without providing justifications. Audio-recorded files will also be kept in folders of the principal investigator’s personal computer and will be password-protected to safeguard the confidentiality of the participants’ details. For the best interest of the participants, both the site (home, workplace, or any other place) and the timing of the interview will be decided by the participants to ensure their privacy and freedom.

The output of the research will be disseminated through academic articles published in international peer-reviewed scientific journals. The participants will be notified regarding the sharing of findings to the public domain; however, confidentiality, anonymity (by providing pseudonyms), and privacy of the participants will be ensured. Our research findings will be reported following the COREQ (Checklist for Reporting Qualitative Research) guidelines [31].

The study protocol was reviewed and approved by Mahidol University Central Institutional Review Board (MU-CIRB 2020/217.1808), Thailand, in September 2020, and the Nepal Health Research Council (716/2020 PhD), Nepal, in January 2021.

## Results

The fieldwork started in February 2021 and different key informants and stakeholders at international and nongovernmental organizations, transplant units, policymakers, government workers, and legal workers have been identified. The data analysis was completed in September 2021.

## Discussion

### Projected Significance

Kidney selling is a global phenomenon; however, concentrated selling is rare and warrants urgent attention. The selling of kidneys from one particular village calls for an in-depth and broad exploration of the issue. The few previous studies on the topic have identified social responsibilities such as education, social welfare, dowry, and debt, entrenched in the local social and cultural context, to be crucial drivers of kidney selling [4,6,10-12]; however, these factors alone may not explain the concentrated selling of kidneys in one particular village in Nepal. This research will delve into the factors affecting kidney selling in this village at the micro and macro levels, and seek to answer why and how such a phenomenon is so prevalent in this village. The findings of this study may ultimately help to inform the policy and interventions to reduce organ trafficking in Nepal.

### Study Limitations

This study will be conducted particularly in a village with a specific population in rural Nepal, which shows unique characteristics; hence, the data obtained may not be generalizable to other populations inside and outside Nepal. The character and the situation of Nepal and its open border with India may bring about findings that may or may not apply to other parts of the world where kidney selling is found.

### Conclusion

This research will explore the reasons for selling kidneys among the residents of Hokse village, which is also known as “the kidney village” in Nepal. This work also attempts to understand the motivations of the individuals from this particular village, including their habits, economic status, employment status, educational attainment, daily activities, behavioral patterns, and understanding of the outer world. We will attempt to determine the social status of the kidney sellers within the village from both sides, kidney sellers and villagers, and to explore whether they are discriminated against or stigmatized in their community. The findings from this research will highlight the characteristics of the phenomenon in the village, including its drivers at micro and macro levels. Future research can build on the findings from this research, including operational and implementation research aiming to mitigate organ trafficking. Findings from this study will help us to establish and challenge our hypothesis and assumptions related to kidney selling in the study village. Moreover, this study can serve as a foundational platform to explore the strands and areas that might emerge but may not have adequate explanatory data. Future research can build from these findings to explore questions and areas that are beyond the scope of the current research.

## Appendix

Multimedia Appendix 1 Interview guide.

## Abbreviations

CMA: critical medical anthropology
COREQ: Checklist for Reporting Qualitative Research
LMIC: low- and middle-income country
