# Supplementary material for: Kidney Sellers From a Village in Nepal: Protocol for an Ethnographic Study
Source: JMIR Res Protoc. 2022 Feb 24;11(2):e29364. doi: 10.2196/29364 (PMC8914735; doi:10.2196/29364)
Supplement: Multimedia Appendix 1 [file resprot_v11i2e29364_app1.pdf]

Supplementary Table 1: Interview Guide

Item	Approach/Guidelines	Researchers' interpretation
1.	**Ethnography** (It will be carried out in the Hokse Bazar)	Ethnography will have observation and perception of the researcher.
	1. Problem of the location where we are going to research	
	2. Awareness of research site	
	3. Objective of our research	
	4. Entry to the research site	
	4.1. Find a suitable place to stay in the village	
	4.2. Contact through the person we know	
	4.3 Chit-chat with the local people	
	4.4 Contact the organization such as primary health care, school, temple, police station	
	5. History of local setting and different ethnic people residing in the area	
	6. Participate in the activities of the villagers such as festival, ceremonies and be visible among the villagers	
	7. Know the leaders and political affiliation of the villagers	
	8. Know the different types of caste, religion people follow	
	9. Know the socio-economic status of villagers and employment history of the villagers	
	10. Know the family background of few families from the village and know their kinship character along with their value to important events	
	11. Know the relationship between male-female, female-female, male-male, elder-younger	
	12. Find the demographic data	
**2. Research at the individual and**	**Key informant interview (Chairman of Ward (Village Development Committee), political leaders, religious leaders, teachers, health post in-charge, Female Community Health**	This will cover the individual

**community levels**	**Volunteers, family members, general populations who are eager to express their opinion on the subject.**	and intermediate levels.
	1. Anyone who has sold kidney… (references)	
	2. Opinion about the people who sold a kidney	
	3. Possible reasons for selling	
	4. Status of sellers in the community	
	5. Any issues noticed after selling	
	6. Any contacts how they involve in selling	
	7. Any organization working for sellers	
	8. Situation of the issue	
	9. Measures to tackle the situations	
	**In-depth Interview- Kidney sellers**<br>***Case study- Special cases (if some of the kidney sellers have exceptionally done better after selling or deteriorated extensively after selling.**	After a thorough understanding of the village and we might have to do it repetitively.
	Socio-demographic information<br>Age, sex, education, number of a family member, income, household structure, marital status	
	About the Kidney selling	
	1. Did you intentionally go to sell a kidney?	
	2. When did you sell your kidney?	
	3. Where did you sell your kidney?	
	4. How did you go to the transplant area?	
	4.1 The place to sell	
	4.2 Place stayed during the selling	
	4.3 Mode of travel to selling place	
	4.4 Were you alone or there were other people with you?	
	5. Any explanation by the doctors	

	6. Decision-making to sell	
	7. Feeling after selling	
	8. How many days did you stay at the hospital after the operation?	
	9. Any talk after the operation	
	10. What happened after the operation?	
	11. Anything you got after selling	
	12. Mode of transportation back to home	
	13. When did you tell your family or relatives or friends about selling your organ?	
	Decision for selling	
	14. Main decision to sell your organ	
	15. Who helped you sell?	
	16. Did you get any payment?	
	Experience after selling an organ	
	17. How did you tell your friends, relatives, family member about your selling?	
	Experienced any problem after selling your organ	
	18. Why do you think that you sold your organ?	
	19. Is there any problem with your family after selling it?	
	20. Share your experiences after selling organ, medically, or socially?	
**3. Interviews (Outside Hokse Bazar)**	**Key-Informant Interviews- Previous researcher (The Asia Foundation), Lawyers, Human Rights defenders, Medical workers (Doctors, Nurse, and pharmaceuticals)**	Research at the intermediate level.
	**For the medical workers**	
	1. What is the flow of nephrology patients at your hospital?	
	2. What are the ratios of OPD to in-patients?	
	3. What are the age groups for the patient admitted?	

	4. What proportion of patients is male and female?	
	5. How is the trend of organ donation over the years?	
	Transplantation seems to have started well in Nepal.	
	6. Is there a special nursing unit for transplant patients?	
	7. Is there a dialysis unit at the hospital?	
	8. How regular are they? Is it free?	
	9. How about transplantation?	
	10. How many organs are transplanted at this facility on a monthly or yearly basis?	
	11. Where do transplanted organs come from? What is the source of transplanted organs?	
	There is news that people from other countries do come to Nepal for transplantation because of equally good services in Nepal comparing with other countries.	
	12. Are medical facilities available for medical tourism?	
	13. How many foreign patients/organ recipients do the institution/facility treat?	
	14. What is the average length of stay in the institute/facility for organ recipients?	
	15. What post-operative care is given to organ donors and recipients? Over what period?	
	16. What is the process of evaluating a potential donor’s eligibility for donation?	
	17. What ethical review procedures are in place?	

	18. How long do organ donors remain in the facility after his or her organ is removed?	
	19. Do organ recipients pay for the organ they receive?	
	20. Have there been any cases or possible of trafficking in persons for the purpose of organ removal?	
	21. Is the institute/facility obliged to report instances of suspected organ trafficking or trafficking in persons for the purpose of organ removal?	
	22. Do you talk to potential donors before transplantation?	
	23. Does the potential donor understand the nature, process, and consequences of organ removal?	
	24. Does the donor fully understand the risks of the procedure?	
	25. Do you do medical checks with the donor before surgery?	
	**For lawyers and Human rights defender**	
	1. Is it legal for living persons to donate organs?	
	2. Is payment for organs illegal?	
	3. Is it possible to receive compensation for organ donation?	
	4. Can non-relatives of the recipient donate organs?	
	5. What other criteria must be met to allow the donation of living organs from living persons?	
	6. Does the law criminalize trafficking in persons? Which law addresses the issue?	
	7. Does trafficking in person's legislation also address/criminalize trafficking for the purpose of organ removal?	
	8. Can medical/health care practitioners be held liable for willful blindness/failure to report?	
	9. Do penalties apply in the event of selling and buying of organs?	

	10. Is there an explicit prohibition of trafficking in persons for organ removal (and also organ trafficking) in the legal framework?	
	11. What are the criteria for consent for organ donation from a living donor?	
	12. What other relevant government and non-government institutions, committees, and/or other bodies are involved in organ donation and transplantation? What is their role?	
	13. How many prosecutions have been laid for trafficking in persons for the purpose of organ removal?	
	14. How many victims of trafficking for the purpose of organ removal are identified each year?	
	15. How many rescued victims of trafficking in persons for the purpose of organ removal have given evidence against their traffickers?	
**4. Observation and interview**	**Key-Informant Interviews-**<br>**Border sites (Observation and interview with legal and social workers)**	
	**Border points**	
	1. What are the duties you have to perform at the border check-post?	
	2. How do people notify about their travel across the border?	
	3. How do you identify the victims?	
	4. What are the works you do to prevent trafficking?	
	5. How many prosecutions have been laid for trafficking in persons for the purpose of organ removal?	
	6. How many victims of trafficking for the purpose of organ removal are identified each year?	
	7. How many rescued victims of trafficking in persons for the purpose of organ removal have given evidence against their traffickers?	
	**Policy Makers and stakeholders- NGO/INGO (Aarogya Foundation, Kathmandu**	

**5. Interview with appointments**	**https://www.facebook.com/aarogya.foundation), MoPH officers, Government officer**	Research at the Macro level.
	1. Actors involved in organ trafficking.	
	2. Modes of trafficking.	
	3. Profiles of organ traffickers and victims.	
	4. Roles of fraud and document falsification.	
	5. Roles of government to stop the act.	
	6. Knowledge of trafficking in persons among investigators, prosecutors, and judges.	
	7. How do we increase awareness about the organ trafficking	
	8. Laws are in place.	
	9. Any successful cooperation or achievements	
	10. Any collaboration with an international agency to track down the cases. Which country?	
	11. Lacking information at your department to respond to trafficking in persons for organ removal.	
	12. What special challenges are involved in prosecuting trafficking in persons cases?	
	13. Has your unit been able to meet these challenges? How? To what level of success?	
	14. What kinds of training, background knowledge, or resources should be made available to law enforcement, immigration officials, prosecutors, and judges to strengthen their response to trafficking in persons for the purpose of organ removal?	
	15. What are the key challenges in addressing trafficking in persons for the purpose of organ removal in the country or context in which you work?	
